# When Interactivity Does Not Directly Trigger Empathy: How Interactive Narrative Experience Shapes Multidimensional Player Empathy in Online Murder Mystery Games

**DOI:** 10.3390/bs16091520

**Published:** 2026-08-28

**Authors:** Junyu Zhu, Wei Wei, Dongning Li

**Affiliations:** 1 School of Design, Jiangnan University, Wuxi 214122, China; 6201609015@stu.jiangnan.edu.cn; 2 4I Design Lab, Suzhou University of Technology, Changshu 215500, China; doublewei@cslg.edu.cn; 3 School of Digital Technology and Creative Design, Jiangnan University, Wuxi 214122, China

**Keywords:** interactive narrative experience, narrative transportation, empathy, Online Murder Mystery Games, perceived interactivity

## Abstract

Interactive narrative experience has become a dominant form of contemporary narrative consumption. Because of its real-time participatory nature, it is widely regarded as an important means of eliciting empathy. Although prior studies have examined the net effects of interactive narrative experience on empathy, they have largely overlooked the configurational pathways associated with empathy formation. Using Online Murder Mystery Games (OMMGs) as the research context, this study surveyed 570 players to examine the relationships among perceived interactivity, perceived narrativity, perceived integrity, narrative transportation, and multidimensional empathy. Empathy was conceptualized as affective empathy, cognitive empathy, and associative empathy, and the study adopted an integrated PLS-SEM and fsQCA approach. The findings show that perceived interactivity, despite long being considered a key driver of empathy, does not directly influence player empathy in OMMGs and only becomes important under specific conditions. In contrast, narrative transportation exerts a significant net effect on empathy and consistently serves as a core condition across high empathy pathways and their subdimensions, suggesting that it plays a more consistently important role than perceived interactivity across the identified configurational pathways. Furthermore, overall empathy and its subdimensions exhibit differentiated configurational patterns, with affective empathy being associated with a more comprehensive configurational pattern than cognitive empathy and associative empathy. The paper develops the theoretical framework, reports the empirical findings, and discusses their theoretical and practical implications.

## 1. Introduction

With the development of interactive media and digital technologies, narrative consumption has gradually shifted from passive reception to active participation (Heravi, 2025). Unlike traditional narrative media such as newspapers, magazines, and films, in which audiences passively receive predetermined narrative information, interactive narrative media such as video games, social platforms, and virtual reality allow users to autonomously intervene in narrative processes through feedback and choice (Koenitz, 2023; Meakin, 2024). As a result, interactive narrative has become one of the most influential forms of contemporary storytelling. Previous studies have shown that interactive narratives play an important role in shaping users’ psychological immersion, emotional engagement, and social cognition (Hao et al., 2025; Polydorou, 2024; Turner, 2025). Among them, interactive narrative games, due to their representativeness and popularity, have become a key research context for examining users’ psychological responses during interactive narrative experiences (Daneels et al., 2023; Toh, 2023; D. Wu et al., 2024).

Online Murder Mystery Games (OMMGs), also known as online Jubensha, are highly popular multiplayer interactive narrative games in contemporary China (D. Wu et al., 2024). Through applications such as WoShiMi (version 3.50.10), BaiBianDaZhenTan (version 6.44.0), and Who Is the Murderer (version 2.1.9), players remotely assume scripted character roles and gradually reconstruct and solve fictional murder cases by reading scripts, collecting clues, and engaging in interactive communication (Xiong et al., 2023). Unlike Branching Narrative Games and Role-playing Games, which primarily rely on predetermined interactive narrative nodes, OMMGs grant players substantial control over the narrative process. Rather than selecting from predefined narrative options, players actively participate in the collaborative construction of story content (Meakin, 2024; Polydorou, 2024). Consequently, OMMGs provide players with a highly open and participatory interactive narrative experience. More importantly, players are particularly likely to develop multidimensional empathy during OMMG experiences (Liang et al., 2025). This is largely because the core gameplay of OMMGs centers on players performing interactive narratives through online role-playing (Yu & Zhang, 2023). On the one hand, players are required to empathize with the characters they portray by understanding their emotions, cognitive perspectives, and behavioral motivations (Y. Liu, 2023). On the other hand, players must also empathize with characters portrayed by other participants by understanding their thoughts and feelings through interactive communication. Therefore, by combining script enactment with role-playing, OMMGs provide an ideal empirical context for exploring how interactive narrative experience shapes players’ multidimensional empathy.

Existing research has explained empathy formation in narrative experiences from multiple theoretical perspectives, including interactive narrative (Meakin, 2024; Ryan, 2006), narrative transportation (Green & Brock, 2000; X. Wu et al., 2024), narrative persuasion (Adornetti et al., 2026), and character identification (Felnhofer et al., 2023). Prior studies have consistently shown that interactive narrative experience elements such as perceived interactivity (PIN), perceived narrativity (PNA), and perceived integrity (PIG) can influence empathy to varying degrees (Adornetti et al., 2026; Gu et al., 2022; Tseng & Thiele, 2024). Among these factors, interactivity, as a core feature distinguishing interactive narratives from traditional narratives (Heravi, 2025), has long been regarded as a key factor directly triggering user empathy (Cuadra et al., 2024; Felnhofer et al., 2023). In addition, narrative transportation (NT) arising from narrative experiences has also been widely considered an important mechanism through which empathy emerges in interactive narrative contexts (X. Wu et al., 2024).

Although existing studies have laid the foundation for understanding empathy among OMMG players, they have primarily focused on the linear net effects between variables, thereby overlooking the complexity of how multiple experiential factors jointly shape empathy and failing to explain the multiple equivalent pathways leading to high empathy. In other words, current research has paid greater attention to whether a specific factor influences empathy, while giving less attention to how different factors combine to produce high empathy. In reality, player empathy in OMMGs is often jointly driven by multiple influencing factors. Moreover, empathy itself consists of multiple dimensions, including affective empathy (AFE), cognitive empathy (CE), and associative empathy (ASE) (Jami et al., 2024), and the formation conditions of these dimensions may not be identical (Ho & Wong, 2026). Therefore, although structural equation modeling can evaluate the net effects of individual latent variables on empathy, it is insufficient for revealing how different variables jointly combine to generate high empathy or how the configurational conditions differ across empathy dimensions in OMMGs.

To address these issues, this study integrates symmetric and asymmetric perspectives to examine how interactive narrative experience shapes multidimensional player empathy in OMMGs and to clarify the net-effect relationships and configurational patterns associated with this process.

Specifically, this study develops a research model grounded in interactive narrative theory (Ryan, 2006), narrative transportation theory (Green & Brock, 2000), and empathy theory (Shen, 2010). It employs a mixed-method approach integrating Partial Least Squares Structural Equation Modeling (PLS-SEM) and fuzzy-set Qualitative Comparative Analysis (fsQCA). PLS-SEM is employed to test the direct effects of perceived interactivity, perceived narrativity, perceived integrity, and narrative transportation on empathy, thereby identifying the symmetric net-effect relationships among the key constructs. FsQCA is used to identify multiple configurational pathways leading to high overall empathy and its three subdimensions, namely affective empathy, cognitive empathy, and associative empathy, thereby revealing the asymmetric pathways underlying empathy formation.

Accordingly, this study addresses the following research questions:

RQ1: How do perceived interactivity, perceived narrativity, and perceived integrity influence narrative transportation and player empathy in OMMGs?

RQ2: Does narrative transportation mediate the relationship between interactive narrative experience and player empathy in OMMGs?

RQ3: What configurational pathways lead to high overall empathy among OMMG players?

RQ4: Do the configurational pathways leading to high affective empathy, cognitive empathy, and associative empathy differ from one another?

By addressing these research questions, this study is expected to make three main contributions. First, it extends theories of interactive narrative, narrative transportation, and empathy into the highly collaborative context of OMMGs, a relatively underexplored genre of interactive media. Second, it provides a more comprehensive understanding of empathy formation by jointly examining overall empathy and its multidimensional structure through complementary symmetric and asymmetric analytical perspectives. Finally, by integrating symmetric and asymmetric analytical approaches, this study provides a more comprehensive perspective for understanding psychological effects in interactive narrative contexts.

## 2. Theoretical Foundations and Hypotheses Development

### 2.1. Online Murder Mystery Game

**Figure 1:**
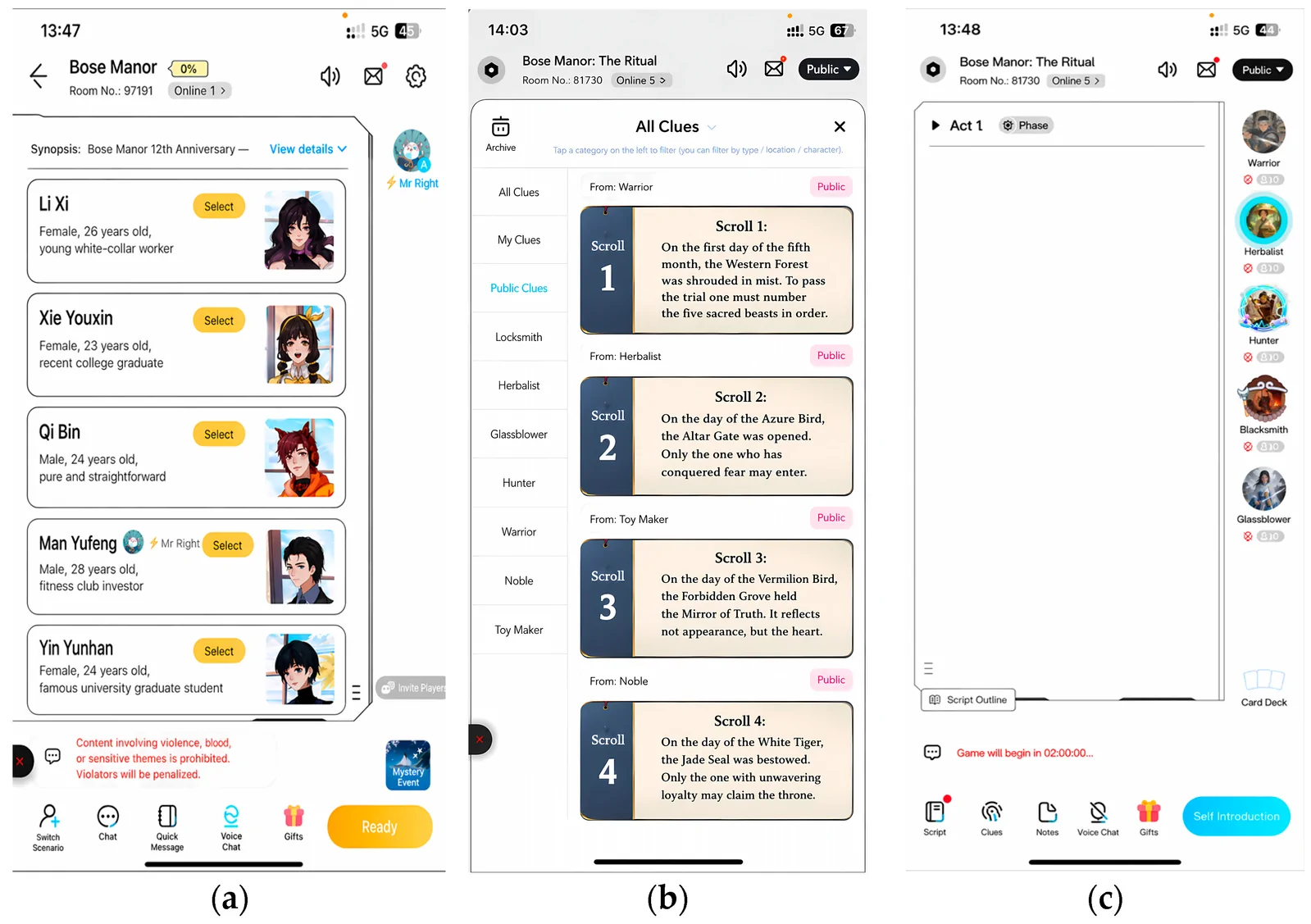
Interface screenshots of the WoShiMi app as an example of OMMGs: (**a**) character selection interface; (**b**) clue collection interface; and (**c**) script reading interface. Source: Screenshots captured by the author from the WoShiMi app.

OMMGs refer to a digitally mediated form of role-playing interactive narrative game in which players participate remotely through mobile applications or online platforms (Liang et al., 2025; Y. Liu, 2023). In these digital environments, players enact specific character roles online, collect clues, engage in dialogue with other participants, and collaboratively solve fictional cases. Compared with traditional offline murder mystery games, OMMGs transform face-to-face interaction into digital forms of online communication, including text, voice, and image-based interaction, thereby enabling remote gameplay experiences (as shown in Figure 1). 

Importantly, OMMGs retain the core narrative mechanisms of traditional murder mystery games. Their narrative structure still revolves around fictional murder cases in which players assume the roles of murderers, suspects, and detectives (Nonomura & Mori, 2025). At the same time, OMMGs preserve key gameplay elements such as script enactment, clue collection, group discussion, deductive reasoning, and murderer identification through online interaction (Xiong et al., 2023). Through these processes, players collaboratively construct narrative content in digital environments. Therefore, OMMGs are not merely online social games, but also digital platforms for interactive narrative creation.

During OMMG experiences, players are required to read narrative texts to understand the settings, identities, and background stories of the characters they portray. Narrative texts occupy a central role in OMMGs because they establish the foundation of the game’s storyline and serve as a key factor in attracting player interest (Yu & Zhang, 2023). Green and Appel (2024) proposed that individuals are highly likely to enter a state of narrative transportation while reading narrative texts. This also applies to OMMGs, particularly because the narrative texts in OMMGs are often more accessible and engaging for players.

After becoming familiar with their character settings, players are required to interact with other participants from the perspectives of the characters they portray. Through these interactions, players exchange character-related information and collaboratively enact the overall narrative content (Ng, 2025; Xiong et al., 2023). During this process, players may develop multiple forms of empathy. First, players may empathize with the characters they portray because they are required to consistently act from the perspectives of those characters throughout the game (Yu & Zhang, 2023). Second, players may empathize with characters portrayed by other participants, as these characters collectively contribute to the unfolding of the narrative event. In addition, players may also develop empathy toward the groups or worlds depicted in the script, as role-playing often leads them to perceive themselves as part of the story world. Taken together, interactive narrative experiences in OMMGs may evoke narrative transportation and multidimensional empathy, thereby contributing to changes in players’ emotions, cognitions, or attitudes (Liang et al., 2025).

### 2.2. Interactive Narrative Experience

Interactive narrative refers to a narrative form in which audiences can actively influence the progression, presentation, or outcome of a story through their actions (Koenitz, 2023; J. Wang, 2025). In OMMGs, players’ actions not only trigger branching dialogues and events but also shape story development and final outcomes. Therefore, players engage in a typical interactive narrative experience in OMMGs. Ryan (2006) argued that interactive narrative is mainly shaped by users’ modes of participation, the structure of interactive nodes, and story integrity. Gu et al. (2022) further identified perceived interactivity, perceived narrativity, and perceived integrity as three first-order dimensions for assessing users’ subjective experience of interactive narratives. Following this framework, the present study systematically assesses players’ interactive narrative experience in OMMGs by measuring their perceived interactivity, perceived narrativity, and perceived integrity.

In this study, perceived interactivity refers to the extent to which players can smoothly obtain relevant narrative information and engage in real-time interaction with other players in OMMGs (Kang et al., 2024; F. Wang et al., 2025). Perceived narrativity refers to players’ overall evaluation of the narrative motivation, emotional construction, and plot continuity of script content in OMMGs (Green & Appel, 2024; Hamby et al., 2024). Perceived integrity refers to the extent to which players perceive the overall story of the script in OMMGs through character settings, story clues, and player communication (Gu et al., 2022; Ryan, 2006).

Numerous studies have shown that interactive narrative experience can positively promote audience narrative transportation (Chen et al., 2026; Thomas & Grigsby, 2024; Zhou & Kim, 2023). On the one hand, this effect stems from the interactive characteristics of interactive narratives. Audiences can control story development in real time through interaction and participate in the construction of the narrative world, thereby entering a state of narrative transportation (Green et al., 2004; Zhou & Kim, 2023). On the other hand, interactive narrative experience contains explicit story elements. Story elements are a necessary condition for narrative transportation, and information presented in story form is particularly likely to elicit narrative transportation (Thomas & Grigsby, 2024). In addition, the coherence and completeness of story plots are also considered important factors that facilitate narrative transportation (Green, 2021; Roth, 2019). Based on these arguments, this study proposes the following hypotheses:

**H1.**  *In Online Murder Mystery Games, perceived interactivity has a positive influence on narrative transportation.*

**H2.**  *In Online Murder Mystery Games, perceived narrativity has a positive influence on narrative transportation.*

**H3.**  *In Online Murder Mystery Games, perceived integrity has a positive influence on narrative transportation.*

In the process of interacting with characters, audiences may develop deep emotional connections with them (Lu & Moller, 2026). They may see aspects of themselves in these characters and resonate with their experiences (Green, 2021), thereby shaping their emotional and cognitive responses (Green & Jenkins, 2014; Ryan, 2015). Accordingly, several studies have shown that interactive narratives can positively shape audience empathy (Hadjipanayi et al., 2024; Ho & Wong, 2026; Tseng & Thiele, 2024). This effect stems not only from the positive role of perceived interactivity in fostering empathy (Li & Sun, 2025), but also from the capacity of stories to allow individuals to indirectly experience others’ lives and thereby cultivate empathy (Hadjipanayi et al., 2024). At the same time, narrative integrity can improve narrative quality and may also positively influence individual empathy. Therefore, this study proposes the following hypotheses:

**H4.**  *In Online Murder Mystery Games, perceived interactivity has a positive influence on empathy.*

**H5.**  *In Online Murder Mystery Games, perceived narrativity has a positive influence on empathy.*

**H6.**  *In Online Murder Mystery Games, perceived integrity has a positive influence on empathy.*

### 2.3. Narrative Transportation and Empathy

The concept of narrative transportation originated from Nell and Nell (1988), who used the metaphor of travel to describe readers’ immersive experience during reading. It was later defined as a distinctive psychological process in which readers enter the narrative world through the integration of attention, imagery, and emotion (Green & Appel, 2024; Green & Brock, 2000). In this state, readers temporarily disengage from the real-world context, such as by ignoring surrounding events or overlooking discrepancies between the narrative and reality (Thomas & Grigsby, 2024). In this study, narrative transportation refers to the extent to which players are transported from the real-world context into the fictional story world during OMMG experiences.

Empathy was initially understood in a relatively simple sense as sharing the emotions of others. However, it has since been developed into a complex, broad, and multidimensional concept (Jami et al., 2024). Therefore, in interactive narrative contexts, empathy should be regarded as a multidimensional psychological construct.

Most studies divide empathy into two components: affective and cognitive empathy (de Waal & Preston, 2017; Preston et al., 2020). Affective empathy refers to the ability to understand and share the emotions of others, i.e., the cognitive and affective processes involved in perceiving and experiencing the emotions of others (Huang et al., 2025). Cognitive empathy refers to the ability to take the perspective of others, recognize, understand, and adopt their viewpoints (Golab & Barbot, 2024; Preston et al., 2020). In fact, dividing empathy into emotional and cognitive components may overlook the interconnectedness between them, and there may be some transformational relationships or mediating structures between affective empathy and cognitive empathy (Preston et al., 2020). Regarding this issue, Shen (2010) proposed that in the state empathy model of information processing, in addition to affect and cognition, association is also an important component that bridges emotional and cognitive communication. Associative empathy refers to identification, where the receiver of information generates a sense of personally experiencing the information event through receiving and understanding the information (Ho & Wong, 2026; Shen, 2010). Conceptually, associative empathy differs from affective empathy, which focuses on emotional sharing, and cognitive empathy, which focuses on perspective-taking, by emphasizing psychological identification with the narrative event and a sense of personal involvement in the fictional experience.

Based on this view, the present study conceptualizes empathy as a reflective–reflective higher-order construct composed of affective empathy, cognitive empathy, and associative empathy (T. Liu et al., 2025; Preston et al., 2020; Shen, 2010). In this study, affective empathy refers to the extent to which players understand and share the emotions of characters and other players during OMMG experiences. Cognitive empathy refers to the extent to which players think from the perspectives of characters and other players, and recognize, understand, and adopt their viewpoints during OMMG experiences. Associative empathy refers to the extent to which players develop a sense of personally experiencing the fictional event by understanding relevant narrative information during OMMG experiences.

Many studies have indicated that narrative transportation is an important mediator between narrative experience and individual emotional change, often leading to changes in emotions, empathy, and attitudes (Thomas & Grigsby, 2024). For example, Cheng (2023) found that, in pedagogical immersive virtual reality, narrative transportation mediated the relationship between epistemic curiosity and cognitive attitudinal learning. Likewise, J.-X. Liu (2023) demonstrated that narrative transportation mediated the relationship between environmental narrative experiences and pro-environmental intentions by enhancing empathy with nature. More recently, Tzeng et al. (2025) further demonstrated that narrative transportation helped translate food involvement and place attachment into more favorable perceptions of destination food among viewers of food-themed films. Empathy means that story receivers attempt to understand the experiences of story characters and perceive and feel the world in ways similar to those characters (J.-X. Liu, 2023). When individuals enter a state of narrative transportation, their self-awareness may be temporarily replaced by the emotions and cognitions of characters, thereby generating strong identification and empathy toward those characters (Eekhof et al., 2023; Green & Appel, 2024; Green & Brock, 2000). For example, Felnhofer et al. (2023) demonstrated experimentally that narrative transportation significantly predicts readers’ identification with the story protagonist. Therefore, this study proposes the following hypothesis:

**H7.**  *Narrative transportation has a positive influence on empathy.*

**Figure 2:**
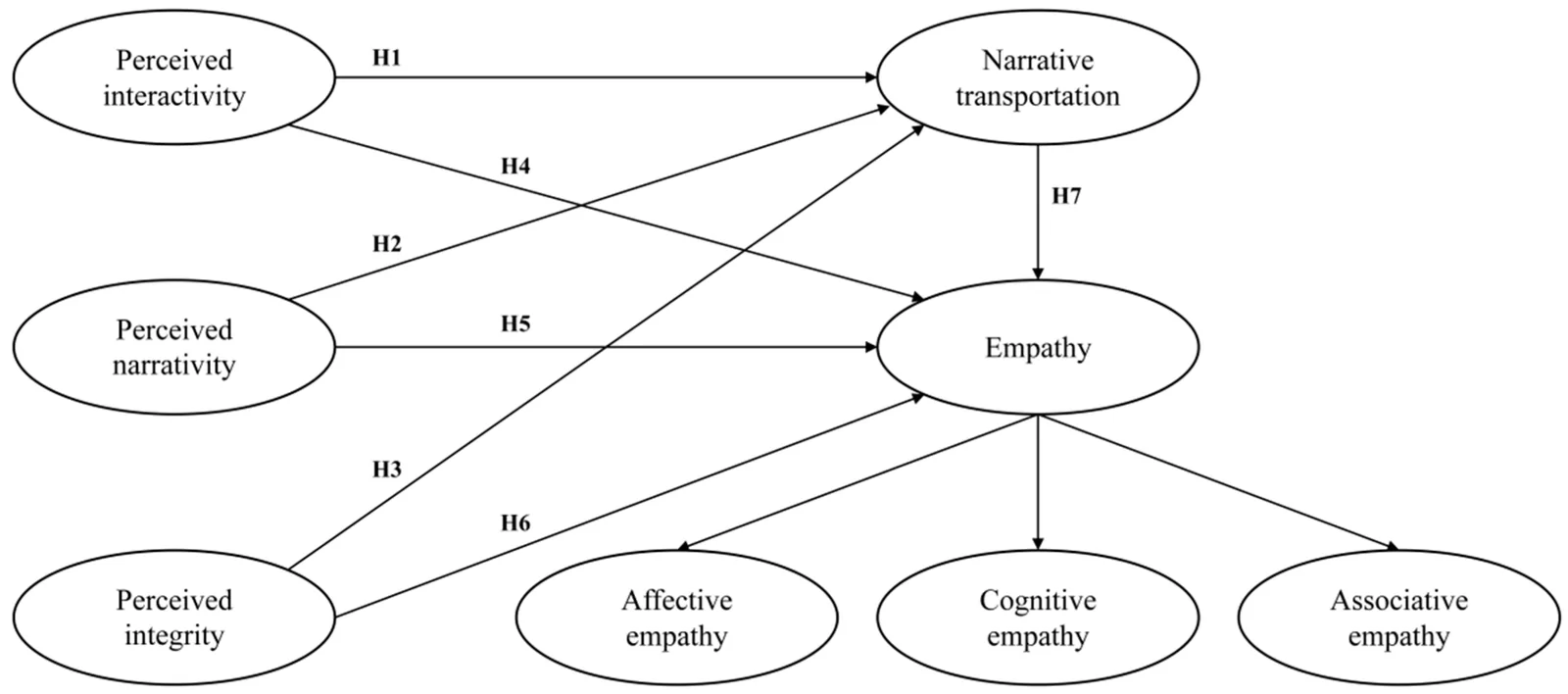
Research model.

In summary, this study aims to examine how interactive narrative experience in OMMGs shapes player empathy and to analyze the mediating role of narrative transportation in this process. The specific research model is shown in Figure 2. Although the above hypotheses emphasize the net effects of perceived interactivity, perceived narrativity, perceived integrity, and narrative transportation on player empathy, empathy formation may also depend on the joint effects of multiple conditions. Therefore, further examination through configurational analysis is necessary.

## 3. Methodology

### 3.1. Questionnaire Design

The survey instrument used in this study consisted of four sections. The first section provided a brief introduction to the basic concept and gameplay mechanisms of OMMGs to ensure that all participants had a consistent understanding of this game form. The second section included a screening question, “Have you ever played an Online Murder Mystery Game?” (yes/no), to identify participants with relevant experience. Respondents who did not meet this criterion were excluded from completing the remaining parts of the questionnaire. The third section collected demographic information, including gender, age, and occupation. The fourth section contained the measurement items for the research constructs, comprising 27 items in total.

This study adopted well-established scales with high reliability and validity from previous research. Perceived interactivity, perceived narrativity, and perceived integrity were measured using 11 items adapted from Gu et al. (2022). Narrative transportation was measured using four items adapted from Sherrick (2021). Empathy, including affective empathy, cognitive empathy, and associative empathy, was measured using 12 items adapted from Augello (2022). All items were measured on a seven-point Likert scale, ranging from 1 = “strongly disagree” to 7 = “strongly agree.”

Given that all participants in this survey were Chinese players, the questionnaire was administered in its finalized Chinese version to ensure accurate comprehension of the items. The questionnaire was translated using a forward–backward translation procedure. Two bilingual researchers fluent in both English and Chinese first translated the questionnaire from English into Chinese. Subsequently, another two bilingual experts who were blind to the original version translated the Chinese version back into English. Wording adjustments were then made through group discussion to ensure semantic equivalence.

Before the final questionnaire was distributed, a pretest was conducted with 46 OMMG players, and the results indicated an acceptable level of internal consistency. In addition, three experts in interactive narrative games were invited to review the questionnaire. Based on their feedback, adjustments were made to the number, order, and wording of the items. The questionnaire was then distributed on a large scale in Chinese. The complete English and Chinese versions of the questionnaire are provided in the Appendix A (see Table A1).

### 3.2. Data Collection

Data were collected online using convenience sampling from OMMG player communities associated with platforms such as WoShiMi, BaiBianDaZhenTan, and Who Is the Murderer. The target sample consisted of Chinese players aged 18 years or older who had prior experience with OMMGs. All participants who completed the questionnaire were eligible to receive a small incentive. Screening questions were placed at the beginning of the survey to ensure that only individuals with prior OMMG experience proceeded with the questionnaire, thereby ensuring alignment between the target population and the research topic. Attention-check items were also embedded in the questionnaire to improve response quality.

Before completing the questionnaire, respondents were required to read an informed consent statement and could proceed only after providing explicit consent. To further reduce the potential influence of common method bias, several procedural remedies were implemented during data collection following the recommendations of Podsakoff et al. (2003). Specifically, the survey was conducted anonymously, and respondents were informed that all responses would remain confidential and be used solely for academic research. They were also assured that there were no right or wrong answers and were encouraged to answer honestly based on their actual experiences. These procedural remedies were intended to minimize evaluation apprehension and social desirability bias.

The questionnaire was distributed between December 2025 and March 2026. A total of 650 responses were collected. After excluding 80 invalid responses from respondents without OMMG experience, those who failed the attention checks, and those with excessively short completion times, 570 valid responses remained for subsequent analysis, yielding an effective response rate of 87.69%. This sample size far exceeds the recommended requirement for PLS-SEM (Hair et al., 2019).

As reported in Table 1, 41.75% of respondents had participated in an OMMG once, while 58.25% had participated two or more times, indicating that the sample largely consisted of individuals with relevant gameplay experience. Furthermore, 80.53% of respondents were aged between 18 and 30, and 53.51% were students, which is consistent with the core user base of OMMGs reported by the Huajing Industry Research Institute (2025). Overall, the sample adequately reflects the basic characteristics of OMMG players.

### 3.3. Data Analysis

To obtain a more comprehensive understanding of how interactive narrative experience shapes multidimensional empathy, this study integrated PLS-SEM and fsQCA. Combining these two methods provides a more comprehensive perspective for understanding the relationships between antecedent constructs and outcome constructs (Sukhov et al., 2023). PLS-SEM was used to examine the relationships among interactive narrative features, narrative transportation, and empathy in OMMGs and to predict the effects of each antecedent on player empathy. It enables the estimation of the net effects of individual antecedents and the testing of the proposed hypotheses. However, as a variance-based and variable-oriented approach, PLS-SEM primarily focuses on symmetric relationships among variables and therefore cannot fully explain how different combinations of interactive narrative experience jointly contribute to high empathy.

To overcome this limitation, fsQCA was employed as a complementary analytical approach. Unlike PLS-SEM, which evaluates the independent contribution of individual variables, fsQCA assumes that outcomes are often produced through multiple combinations of conditions rather than a single antecedent. This configurational perspective is particularly suitable for the present study because player empathy in OMMGs is jointly shaped by multiple interactive narrative features and narrative transportation, and its different dimensions may emerge through distinct configurational pathways. Therefore, fsQCA enables the identification of multiple configurations leading to high overall empathy as well as affective empathy, cognitive empathy, and associative empathy, thereby providing complementary insights into the configurational pathways underlying empathy formation.

This integrated approach has been widely applied in user experience research. For example, Zhang et al. (2025) used PLS-SEM and fsQCA to examine whether technology readiness and game immersion experience influence tourists’ willingness to participate in metaverse tourism. Mehrab Ashrafi et al. (2025) applied this mixed-method approach to investigate users’ recommendation behavior toward mobile fitness applications.

Accordingly, this study employs PLS-SEM to examine the symmetric relationships among perceived interactivity, perceived narrativity, perceived integrity, narrative transportation, and multidimensional empathy, and to test the proposed hypotheses. Subsequently, fsQCA is applied to identify multiple configurational pathways leading to high overall empathy as well as affective empathy, cognitive empathy, and associative empathy. By integrating these two analytical approaches, this study provides a more comprehensive understanding of both the symmetric net effects and configurational pathways leading to multidimensional empathy in OMMGs.

## 4. Results

To examine both the net linear relationships and the complex configurational patterns underlying player empathy in OMMGs, this study adopted an integrated PLS-SEM and fsQCA analytical framework. First, PLS-SEM was conducted using SmartPLS 4.1.1.4 to assess the measurement and structural models. Subsequently, fsQCA was conducted using fsQCA 3.0 based on the latent variable scores exported from SmartPLS. The fsQCA first explored configurational pathways leading to high overall empathy as a second-order construct and then further examined the distinct causal combinations associated with high affective empathy, cognitive empathy, and associative empathy. This procedure provides a more fine-grained understanding of how player empathy is formed.

### 4.1. PLS-SEM Results

Because empathy was conceptualized as a reflective–reflective higher-order construct composed of affective empathy, cognitive empathy, and associative empathy, the Two-Stage Approach was employed for the PLS-SEM analysis (Sarstedt et al., 2019). Specifically, the PLS-SEM results are reported in three stages. First, the measurement model of all first-order constructs was assessed to establish construct reliability and validity and to obtain the latent variable scores required for the higher-order construct. Second, the second-order measurement model incorporating empathy was evaluated. Finally, the structural model was tested to examine the hypothesized relationships and their statistical significance.

#### 4.1.1. Measurement Model Assessment

As shown in Table 2, all first-order constructs satisfied the recommended threshold values proposed by Hair et al. (2019). All indicator loadings ranged from 0.770 to 0.890, exceeding the recommended value of 0.70 and indicating satisfactory indicator reliability. All VIF values were below 3.3, suggesting a low likelihood of common method bias (Kock, 2015). Cronbach’s α and composite reliability (CR) values ranged from 0.810 to 0.889 and from 0.888 to 0.923, respectively, demonstrating adequate internal consistency reliability. The average variance extracted (AVE) values ranged from 0.675 to 0.750, all above the recommended value of 0.50, confirming satisfactory convergent validity.

Following the two-stage approach, the latent variable scores of AFE, CE, and ASE were used as lower-order constructs (LOCs) to estimate the higher-order construct (HOC) of empathy. As shown in Table 3, the loadings of the three LOCs ranged from 0.895 to 0.923, indicating that each dimension robustly represented the higher-order empathy construct. The corresponding VIF values ranged from 2.520 to 3.094, falling below the threshold of 3.3 and suggesting no critical collinearity issues. The Cronbach’s α, CR, and AVE values for the HOC were 0.897, 0.936, and 0.830, respectively, all exceeding the recommended thresholds. These results confirm that empathy demonstrated satisfactory reliability and convergent validity as a reflective–reflective second-order construct. This finding supports the conceptualization of empathy as a multidimensional construct reflected by affective empathy, cognitive empathy, and associative empathy and justifies the use of the higher-order construct in the subsequent PLS-SEM and fsQCA analyses.

Following the establishment of empathy as a higher-order construct, discriminant validity of the final measurement model was further assessed using the Fornell–Larcker criterion and the heterotrait–monotrait ratio (HTMT). As shown in Table 4, the square root of the AVE for each construct exceeded its correlations with other constructs, satisfying the Fornell–Larcker criterion. In addition, the HTMT values ranged from 0.657 to 0.874, all remaining below the recommended threshold of 0.90 (Henseler et al., 2015). These findings indicate an acceptable level of discriminant validity among the constructs. For the higher-order construct (EMP), the square root of AVE (0.911) exceeded its correlations with perceived interactivity (0.622), perceived narrativity (0.692), perceived integrity (0.626), and narrative transportation (0.744). Likewise, the HTMT values between EMP and the other constructs ranged from 0.710 to 0.855, all below the recommended threshold of 0.90, confirming that the higher-order empathy construct is empirically distinct from the other latent constructs. The satisfactory discriminant validity of the measurement model provides a sound measurement foundation for the subsequent PLS-SEM and fsQCA analyses, allowing the respective roles of the latent constructs to be examined without substantial conceptual overlap.

#### 4.1.2. Structural Model Assessment

**Figure 3:**
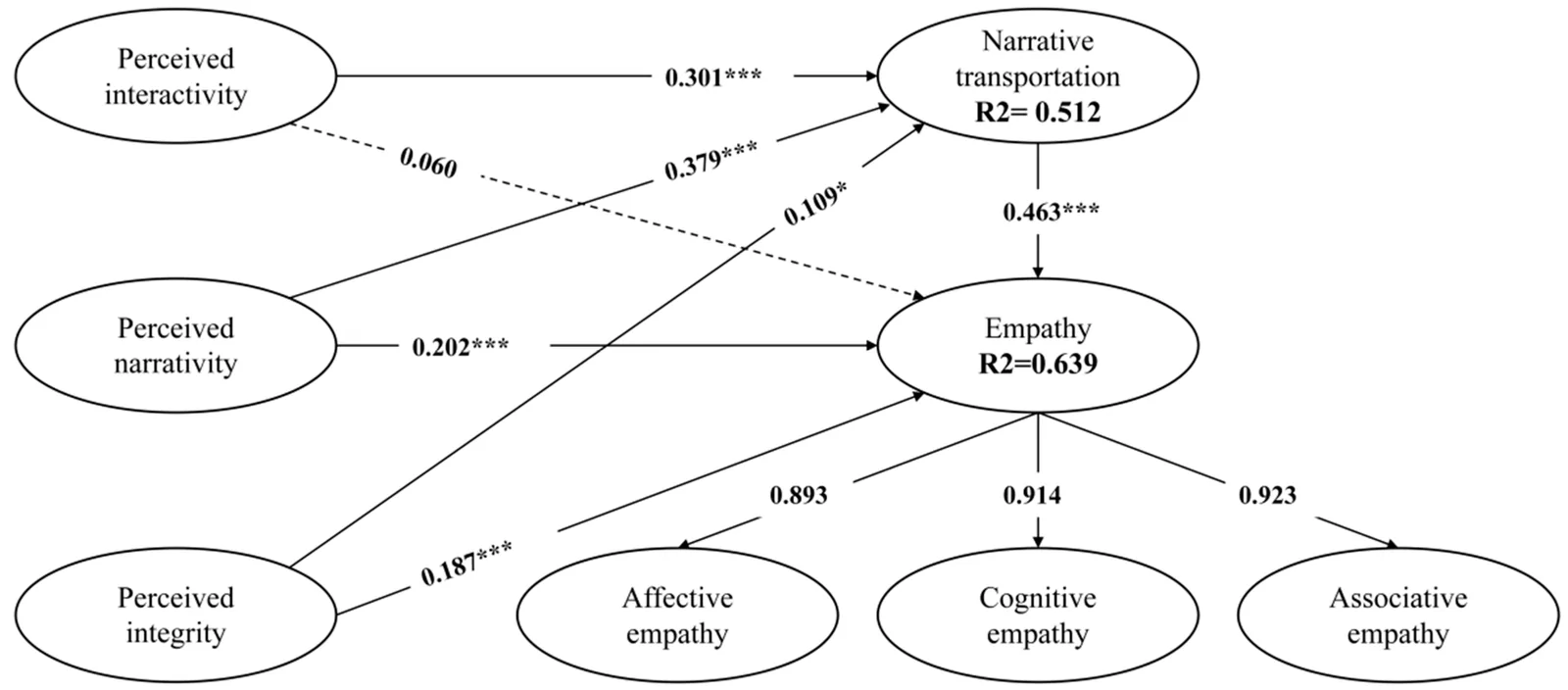
Structural model results. Note: Standardized path coefficients are reported; *** *p* < 0.001, * *p* < 0.05; dashed lines indicate non-significant paths; values between higher-order and lower-order constructs represent outer loadings.

Bootstrapping with 5000 resamples was performed to evaluate the significance of the structural paths at the 95% confidence interval. Figure 3 illustrates the final structural model, and Table 5 summarizes the path coefficients (β), t-values, significance levels (*p*-values), effect sizes (f^2^), coefficients of determination (R^2^), and hypothesis testing results.

The model demonstrated satisfactory explanatory power, with R^2^ values of 0.512 for NT and 0.639 for empathy. In addition, the SRMR value of the estimated model was 0.050, which was below the recommended threshold of 0.08, indicating an acceptable overall model fit (Henseler et al., 2014). The inner VIF values ranged from 2.049 to 3.127, all remaining below the conservative threshold of 3.3, indicating no critical multicollinearity issue within the structural model. Predictive relevance was further confirmed through the PLSpredict procedure, as all Q^2^ predict values were above zero, ranging from 0.308 to 0.459, which indicates adequate predictive capability of the model (Shmueli et al., 2019).

The hypothesis testing results revealed that six of the seven proposed hypotheses were supported, indicating that the proposed research model received substantial empirical support. PIN (β = 0.301, *p* < 0.001), PNA (β = 0.379, *p* < 0.001), and PIG (β = 0.109, *p* < 0.05) all exerted significant positive effects on NT, thereby supporting H1, H2, and H3. These findings indicate that all three dimensions of interactive narrative experience were significantly associated with narrative transportation, with perceived narrativity exhibiting the strongest relationship. For empathy, PNA (β = 0.202, *p* < 0.001), PIG (β = 0.187, *p* < 0.001), and NT (β = 0.463, *p* < 0.001) had significant positive effects, supporting H5, H6, and H7. Among these predictors, narrative transportation exhibited the strongest association with empathy, followed by perceived narrativity and perceived integrity. However, the direct effect of PIN on empathy was not significant (β = 0.060, *p* = 0.102), leading to the rejection of H4. This result indicates that perceived interactivity was not directly associated with player empathy in OMMGs. In terms of effect size, most structural paths exhibited small to medium effects, whereas NT showed the strongest explanatory contribution to empathy (f^2^ = 0.290).

Further mediation analysis was conducted to examine the indirect effects via NT. As shown in Table 6, the indirect effects of PIN (β = 0.139, *p* < 0.001), PNA (β = 0.176, *p* < 0.001), and PIG (β = 0.050, *p* < 0.05) on empathy were all significant. Among them, PNA exhibited the largest indirect effect, followed by PIN and PIG. These results support the mediating role of NT in the relationships between interactive narrative experience and empathy.

### 4.2. FsQCA Results

This study employed fsQCA to explore the configurational pathways leading to high overall empathy and its subdimensions. The analysis was conducted in two stages: first focusing on overall empathy as a second-order construct and then on its three first-order dimensions, namely AFE, CE, and ASE. Following the direct calibration method (anchors: 0.95, 0.50, 0.05), necessity and sufficiency analyses were subsequently performed for each outcome condition. To further examine the robustness of the configurational findings, additional analyses were conducted using alternative calibration anchors (0.90, 0.50, 0.10).

#### 4.2.1. Configurational Pathways Leading to High Overall Empathy

The latent variable scores derived from the second-order measurement model were used for the fsQCA analysis of high overall empathy. These scores were first calibrated into fuzzy-set membership values ranging from 0 to 1 using the direct calibration method. Following Yuan et al. (2024), the 5th percentile, 50th percentile, and 95th percentile were adopted as the thresholds for full non-membership, crossover, and full membership. The calibration breakpoints for each construct are presented in Table 7.

Subsequently, a necessity analysis was conducted to examine whether any single antecedent condition constituted a necessary condition for high overall empathy. As shown in Table 8, the consistency values for PIN, PNA, PIG, and NT, together with their negated forms, ranged from 0.507 to 0.839, all below the recommended threshold of 0.90 (Ragin, 2009). This indicates that neither any individual antecedent condition nor its absence could be considered a necessary condition for high overall empathy. Therefore, a sufficiency analysis was next performed to identify the configurational pathways leading to high overall empathy.

This study constructed a truth table and simplified it using the thresholds of 10 cases for frequency, 0.80 for consistency, and 0.70 for PRI consistency (Pappas & Woodside, 2021). As shown in Table 9, three condition combinations leading to high overall empathy among players were identified. Pathway 1 indicates that high levels of PIN, PNA, and NT can lead to high overall empathy. Pathway 2 shows that the presence of PIN, PIG, and NT can lead to high overall empathy. Pathway 3 suggests that high levels of PNA, PIG, and NT can lead to high overall empathy. By comparing the intermediate and parsimonious solutions, PNA and NT were identified as core conditions in Pathways 1 and 3, while PIN and PIG served as peripheral conditions. In Pathway 2, PIN, PIG, and NT were all identified as core conditions.

In addition, all condition combinations exhibited high consistency values (0.915–0.921) and coverage values (0.646–0.670). The overall solution consistency was 0.900, and the overall solution coverage was 0.744. This indicates that the three configurations effectively explain cases of high player empathy in OMMGs and cover a substantial proportion of cases involving high overall empathy among OMMG players (Pappas & Woodside, 2021; Yuan et al., 2024).

#### 4.2.2. Configurational Pathways Leading to High Empathy Subdimensions

To further examine the configurational differences across empathy dimensions, AFE, CE, and ASE were separately treated as outcome conditions. Using the same direct calibration procedure as in the previous stage, the latent variable scores from the first-order measurement model were recalibrated. The corresponding calibration anchors are shown in Table 10.

Necessity analyses were then conducted for the three empathy subdimensions. The consistency values of all antecedent conditions (PIN, PNA, PIG, and NT) and their negated forms were below the recommended threshold of 0.90, indicating that none of these antecedent conditions constituted a necessary condition for achieving high AFE, CE, or ASE. Therefore, sufficiency analyses were subsequently performed.

Following the same truth table construction and reduction criteria adopted in the previous stage, the configurational solutions for the three empathy subdimensions are presented in Table 11. The results reveal differentiated configurational patterns across empathy dimensions.

Specifically, high AFE was explained by a single sufficient configuration, in which PIN, PNA, PIG, and NT all appeared as core present conditions. In contrast, both high CE and high ASE were generated through two alternative configurations. In these pathways, PNA and NT consistently emerged as core present conditions, whereas PIN and PIG functioned as peripheral supporting conditions. This indicates that both CE and ASE are strongly dependent on players’ PNA and NT, while PIN and PIG serve as peripheral complementary conditions that further strengthen the empathic process through alternative combinations.

All configurational solutions reported consistency values above the recommended threshold of 0.80. The overall solution consistency and coverage were 0.878 and 0.608 for high AFE, 0.899 and 0.699 for high CE, and 0.900 and 0.696 for high ASE, respectively. These findings suggest that although the three empathy subdimensions exhibit differentiated causal structures, the identified configurations all demonstrate satisfactory explanatory adequacy and account for a substantial proportion of high-empathy cases.

Taken together, the PLS-SEM and fsQCA results show both convergence and complementarity in explaining player empathy. Specifically, PNA, PIG, and NT received consistent support across the two analytical approaches, indicating that these factors not only exert significant net effects on empathy but also repeatedly function as important configurational conditions in generating high-empathy outcomes. In particular, PNA and NT emerged as the most stable antecedents, appearing as core present conditions in most high-empathy solutions. By contrast, although PIN did not show a significant direct effect on empathy in the PLS-SEM model, it appeared repeatedly as a core or peripheral condition in several fsQCA pathways. This suggests that PIN may not independently influence empathy in a linear manner, but still plays an important role when combined with other favorable narrative conditions.

#### 4.2.3. Robustness Analysis

To assess the robustness of the fsQCA findings, additional analyses were conducted using alternative calibration anchors (10th, 50th, and 90th percentiles) instead of the original calibration thresholds (5th, 50th, and 95th percentiles). The recalibrated analyses yielded largely consistent configurational patterns for both the overall empathy model and the three empathy subdimensions. Although a minor variation was observed in the associative empathy model, where two original pathways converged into one under the alternative calibration, the core configurational pattern remained substantively unchanged. These results provide additional support for the robustness of the reported findings. Detailed robustness results are presented in Appendix A.2.

## 5. Discussion

It should be noted that, given the correlational and cross-sectional nature of the data, all relationships reported in this study should be interpreted as associations rather than causal effects.

Using OMMGs as the research context, this study enriches current understanding of how multidimensional empathy is formed in interactive narrative experiences by integrating symmetric and asymmetric analytical perspectives. The PLS-SEM results confirm several relatively stable linear relationships identified in prior research. Specifically, perceived interactivity, perceived narrativity, and perceived integrity all exert significant positive effects on narrative transportation (H1, H2, and H3 supported), while perceived narrativity, perceived integrity, and narrative transportation significantly and positively influence player empathy (H5, H6, and H7 supported). These findings indicate, on the one hand, that interactive narrative experience in OMMGs enables players to enter a state of narrative transportation and develop empathy. On the other hand, they show that vivid storylines and complete story information in OMMGs can directly foster player empathy. These findings are consistent with prior research on interactive narrative, narrative transportation, and empathy (Chen et al., 2026; Cheng, 2023; Li & Sun, 2025) suggesting that these theoretical explanations remain applicable in the OMMG context.

More importantly, the combined evidence from PLS-SEM and fsQCA indicates that player empathy in OMMGs cannot be adequately explained by a single factor or simple linear relationships. Instead, its formation reflects a more complex condition-dependent pattern involving the joint effects of different factors and multiple configurational pathways. In particular, several findings warrant further discussion, including the differentiated results concerning perceived interactivity across the two methods, the core role of narrative transportation, and the configurational differences between overall empathy and its subdimensions.

### 5.1. Perceived Interactivity Is Not Universally Associated with Empathy but Becomes Important Under Specific Narrative Conditions

A particularly noteworthy finding of this study is that the role of perceived interactivity in empathy varies across different narrative conditions. Although interactivity has long been regarded as a core feature of interactive narrative experience (Heravi, 2025; Roth, 2019) and is often assumed to directly stimulate users’ emotional engagement and empathic involvement (Cuadra et al., 2024; Felnhofer et al., 2023; Green et al., 2004; Zhou & Kim, 2023), the present findings suggest that this assumption may oversimplify the actual role of interactivity in OMMGs.

Specifically, the PLS-SEM results show that the linear effect of perceived interactivity on overall empathy is not significant (H4 not supported). This indicates that frequent interaction in OMMGs does not necessarily elicit player empathy. One possible explanation is that interaction in OMMGs is unusually frequent and often requires immediate responses, which may leave players with insufficient psychological space for sustained emotional processing and thereby disrupt empathy formation. Throughout the game, players must continuously make conversational choices, interpret others’ statements, conceal or disclose information, and strategically adjust subsequent interactions (D. Wu et al., 2024). This high degree of interactive freedom and continuous demand for response may keep players cognitively focused on “how to interact next,” rather than allowing them enough time to emotionally engage with the suffering, motivations, or tension experienced by narrative characters. In other words, players may be highly engaged at the behavioral level while remaining insufficiently engaged at the emotional level. Prior studies have similarly suggested that excessive interaction may disrupt narrative coherence, weaken the stability of immersive experience, and ultimately reduce emotional engagement (Koenitz, 2023; Ryan, 2015; J. Wang, 2025). In this sense, a high level of perceived interactivity does not necessarily directly activate stronger empathy.

Nevertheless, the mediation analysis indicates that perceived interactivity exerts a significant indirect effect on empathy through narrative transportation. This suggests that the contribution of perceived interactivity to empathy is primarily reflected through narrative transportation rather than through a direct relationship with empathy. Rather than directly enhancing empathy, interactive experiences appear to facilitate players’ immersion in the narrative world, which is in turn associated with higher levels of empathy.

Meanwhile, the fsQCA findings show that perceived interactivity appears as a peripheral condition in Configuration 1 and as a core condition in Configuration 2 for high overall empathy. These findings further suggests that, in OMMGs, the role of perceived interactivity should be understood as conditional rather than universal. Interactivity is not a universally effective linear predictor of empathy. It appears to play an important role only when combined with favorable narrative and psychological conditions, such as stronger narrativity, integrity, or narrative transportation.

The configurational analysis of empathy subdimensions further shows a differentiated role of perceived interactivity. It is a core condition in the only configuration leading to high affective empathy, but only a peripheral condition in one pathway leading to high cognitive empathy and in one pathway leading to high associative empathy. These findings suggest that perceived interactivity appears to be more important for affective empathy than for cognitive or associative empathy. By contrast, cognitive empathy and associative empathy may still emerge through alternative configurational pathways in which perceived interactivity plays a less central role.

Clearly, compared with other empathy dimensions, affective empathy appears to be more closely associated with perceived interactivity in the current narrative context (Tseng & Thiele, 2024). This also supports prior arguments that shared emotional experience is typically grounded in high-quality interaction and shared affective experiences (Szanto & Krueger, 2019).

### 5.2. Narrative Transportation: A Central Psychological Factor Associated with Player Empathy

The PLS-SEM and fsQCA results consistently highlight the central role of narrative transportation in the relationship between interactive narrative experience and player empathy.

From the symmetric analytical perspective, narrative transportation not only has a significant positive effect on overall empathy, with the largest path coefficient, but also serves as a mediator linking perceived interactivity, perceived narrativity, and perceived integrity to player empathy. This means that the effect of interactive narrative experience on empathy does not arise solely from experiential elements themselves. More importantly, it depends on the extent to which these elements transport players into the fictional narrative world (Eekhof et al., 2023).

The asymmetric analysis further reinforces the importance of narrative transportation. As a core condition, narrative transportation appears in all configurations leading to high overall empathy and is also present in all configurational pathways for affective empathy, cognitive empathy, and associative empathy. This stability across overall empathy and its three subdimensions suggests that narrative transportation is not merely one factor among many. Rather, it functions as a shared psychological gateway through which different forms of empathy can be fostered (J.-X. Liu, 2023; Thomas & Grigsby, 2024).

This finding is highly consistent with narrative transportation theory. The theory suggests that when individuals are transported into a narrative world, they are more likely to align emotionally and cognitively with story characters (Green & Appel, 2024; Green & Brock, 2000). In the OMMG context, this mechanism may be even more pronounced because players do not observe the story as external spectators; instead, they become the characters themselves. Through role-playing, clue integration, and collaborative reasoning, players actively participate in the narrative process (D. Wu et al., 2024). Once players are transported into the fictional murder case, they are more likely to temporarily suspend awareness of the real world, adopt the perspectives of their characters, and develop empathy through script enactment. Therefore, what may be more important for empathy is not the surface-level interactive narrative experience itself, but the deeper process of psychological immersion and character involvement (Green, 2021). It is also worth noting that the primary objective of the present study was to examine the relationship between narrative transportation and multidimensional empathy in the overall OMMG player population. Accordingly, the final sample comprised a relatively balanced proportion of male (53.33%) and female (46.67%) players. Nevertheless, this should not be interpreted as evidence that potential gender differences are negligible. Because the present study did not statistically examine whether this relationship differs by gender, the possibility that the strength of the relationship between narrative transportation and multidimensional empathy varies between male and female players cannot be ruled out.

### 5.3. Configurational Connections and Differences Between Overall Empathy and Its Subdimensions

Another important finding from the fsQCA results is that the configurational complexity of empathy differs substantially between overall empathy and its subdimensions. Specifically, high overall empathy can be achieved through three sufficient pathways, whereas affective empathy emerges through only one sufficient pathway. Cognitive empathy and associative empathy each show two alternative pathways. These findings indicate that, in OMMGs, different dimensions of empathy depend on distinct condition combinations rather than sharing a uniform configurational logic.

First, there are clear connections between the configurational structure of overall empathy and those of its specific subdimensions. Among the three pathways leading to high overall empathy, two exhibit a similar configurational logic, characterized by perceived narrativity and narrative transportation as core conditions, with either perceived interactivity or perceived integrity serving as a peripheral condition. This indicates that strong story content and sufficient narrative transportation jointly constitute an important foundation for overall empathy. This driving pattern also applies to the configurational pathways for cognitive empathy and associative empathy. This suggests that overall empathy shares greater configurational similarity with cognitive empathy and associative empathy than with affective empathy.

Second, clear differences emerge between the configurational pathways of overall empathy and those of its subdimensions. Overall empathy is supported by two distinct driving types: one centered on the presence of perceived narrativity and narrative transportation as core conditions, and the other centered on the presence of perceived interactivity, perceived integrity, and narrative transportation as core conditions. In contrast, each empathy subdimension is primarily anchored in one dominant configurational pattern. This suggests that overall empathy, as a broad composite outcome, can be formed through more diverse condition combinations, whereas the activation of specific empathy dimensions requires more targeted narrative conditions.

Finally, among the three empathy dimensions, the configurational pathway associated with affective empathy involves the simultaneous presence of all four core conditions, whereas the pathways for cognitive empathy and associative empathy involve fewer core conditions. High affective empathy emerges through only one sufficient pathway, in which perceived interactivity, perceived narrativity, perceived integrity, and narrative transportation must all be present as core conditions. This suggests that, while players may cognitively understand characters or imaginatively project themselves into the story under more limited narrative conditions, high affective empathy is associated with the simultaneous presence of active interaction, engaging narrative expression, complete informational support, and deep narrative transportation.

Overall, this study finds that empathy formation in OMMGs should be understood as a multidimensional, hierarchical psychological process jointly shaped by multiple factors, rather than as the direct result of any single interactive narrative experience factor.

## 6. Conclusions

Integrating the net effects identified by PLS-SEM with the configurational sufficiency revealed by fsQCA, this study offers three key insights. Specifically, narrative transportation consistently serves as a core configurational condition across different empathy outcomes. Furthermore, other factors, such as perceived interactivity, become influential only within specific favorable combinations of narrative conditions. More importantly, different empathy dimensions exhibit distinct configurational requirements, with affective empathy exhibiting a more comprehensive combination of favorable conditions.

### 6.1. Theoretical and Practical Contributions

This study makes four main theoretical contributions. First, this study demonstrates that many of the relationships identified in prior research on interactive narrative, narrative transportation, and empathy are also observed in OMMGs, while its primary contribution lies in examining these established relationships within OMMGs, a relatively underexplored genre of interactive media. Second, in the OMMG context, this study enriches the existing literature by moving beyond the traditional net-effect perspective to reveal the configurational role of narrative transportation in empathy formation. Third, this study offers an alternative perspective on the conventional view that perceived interactivity directly promotes empathy by emphasizing that, in OMMGs, its role should be understood in conjunction with other dimensions of interactive narrative experience. Finally, this study advances the understanding of multidimensional empathy by comparing the distinct configurational patterns underlying overall empathy and its subdimensions.

The findings of this study also provide practical implications for designers and operators of OMMGs and related interactive narrative platforms. To cultivate player empathy, practitioners should avoid relying solely on the isolated improvement of a single experiential factor. Instead, the configurational findings suggest that empathy is more likely to emerge when narrative, interactive, and narrative transportation-related conditions are jointly optimized. Furthermore, the configurational findings suggest that affective empathy is associated with a more comprehensive combination of favorable conditions than cognitive empathy and associative empathy. Therefore, designers aiming to enhance players’ affective empathy should consider coordinating improvements across the experiential dimensions examined in this study.

### 6.2. Limitations

Although this study identifies several meaningful findings, it also has some limitations. Primarily, the sample was collected exclusively from OMMG players in China using convenience sampling, which may limit the generalizability of the findings to other cultural contexts and narrative environments. In light of this, future research could employ more diverse sampling strategies and cross-cultural comparisons to examine whether the identified configurational pathways remain stable across different countries and narrative settings. Furthermore, this study relied on self-reported questionnaire data collected using a cross-sectional design. Although procedural and statistical remedies were adopted to mitigate potential biases, self-reported data may still be subject to common method bias and other response biases, and the research design cannot completely rule out potential endogeneity, nor can it capture the dynamic evolution of empathy formation during repeated interactive narrative experiences or establish reliable causal relationships among the examined variables. To address this, future research could adopt longitudinal or experimental designs to provide stronger evidence for causal relationships and further examine how empathy develops over time in interactive narrative contexts. Methodologically, although this study integrates symmetric and asymmetric analytical approaches, it did not examine whether the relationships identified in the proposed model vary across different player groups (e.g., gender, age, or prior OMMG experience). Future research could employ multigroup SEM to compare the structural relationships among interactive narrative experience, narrative transportation, and multidimensional empathy across different player groups, as well as combine other methods, such as Necessary Condition Analysis, behavioral tracking, or experimental intervention, to provide stronger evidence for the relationships underlying empathy in interactive narrative environments.

## Figures and Tables

**Table 1:** Sample structural characteristics.

Sample	Category	Number	Percentage
Gender	Male	304	53.33%
	Female	266	46.67%
Age	18–25	340	59.65%
	26–30	119	20.88%
	31–35	74	12.98%
	36–40	32	5.61%
	41+	5	0.88%
Occupation	Students	305	53.51%
	State-owned enterprises	49	8.60%
	Private enterprises	94	16.49%
	Foreign-funded enterprise or multinational corporation	17	2.98%
	Institution or public institution	40	7.02%
	Civil servant or government employee	15	2.63%
	Freelancer or self-employed (Others)	50	8.77%
Preferred level of script difficulty	Beginner	142	24.91%
	Advanced or intermediate	285	50.00%
	Challenging	143	25.09%
Number of times playing Online Murder Mystery Games	Once	238	41.75%
	Twice or more	332	58.25%

**Table 2:** Measurement model analysis results.

Construct	Item	Loadings	VIF	α	CR	AVE
Perceived interactivity	PIN1	0.870	1.946	0.810	0.888	0.725
	PIN2	0.885	2.003			
	PIN3	0.798	1.561			
Perceived narrativity	PNA1	0.868	2.358	0.855	0.902	0.698
	PNA2	0.861	2.274			
	PNA3	0.840	2.007			
	PNA4	0.770	1.634			
Perceived integrity	PIG1	0.864	2.520	0.867	0.910	0.716
	PIG2	0.890	2.812			
	PIG3	0.836	2.013			
	PIG4	0.792	1.661			
Narrative transportation	NT1	0.812	1.888	0.839	0.892	0.675
	NT2	0.871	2.289			
	NT3	0.775	1.651			
	NT4	0.825	1.867			
Affective empathy	AFE1	0.799	1.729	0.853	0.901	0.695
	AFE2	0.861	2.185			
	AFE3	0.866	2.364			
	AFE4	0.806	1.801			
Cognitive empathy	CE1	0.869	2.393	0.887	0.922	0.748
	CE2	0.885	2.691			
	CE3	0.887	2.618			
	CE4	0.817	1.892			
Associative empathy	ASE1	0.854	2.137	0.889	0.923	0.750
	ASE2	0.851	2.212			
	ASE3	0.874	2.519			
	ASE4	0.884	2.616			

**Table 3:** Assessment of the higher-order construct.

Higher-Order Construct	Item	Loadings	VIF	α	CR	AVE
Empathy (EMP)	AFE	0.895	2.520	0.897	0.936	0.830
	CE	0.914	2.773			
	ASE	0.923	3.094			

**Table 4:** Discriminant validity assessment results.

	PIN	PNA	PIG	NT	EMP
**PIN**	**0.852**	0.874	0.725	0.779	0.726
**PNA**	0.735	**0.836**	0.824	0.798	0.788
**PIG**	0.611	0.708	**0.846**	0.657	0.710
**NT**	0.646	0.677	0.561	**0.822**	0.855
**EMP**	0.622	0.692	0.626	0.744	**0.911**

Note: Diagonal elements indicate the square root of AVE values and are bolded; inter-construct correlations are reported below the diagonal; HTMT values are reported above the diagonal. PIN: Perceived interactivity; PNA: Perceived narrativity; PIG: Perceived integrity; NT: Narrative transportation; EMP: Empathy.

**Table 5:** Results of hypotheses testing.

Hypothesis	Path	β	t	*p*	f^2^	R^2^	Result
H1	PIN → NT	0.301	6.135	0.000	0.082	0.512	Supported
H2	PNA → NT	0.379	6.697	0.000	0.104		Supported
H3	PIG → NT	0.109	2.193	0.028	0.012		Supported
H4	PIN → EMP	0.060	1.635	0.102	0.004	0.639	Not supported
H5	PNA → EMP	0.202	4.271	0.000	0.036		Supported
H6	PIG → EMP	0.187	4.838	0.000	0.046		Supported
H7	NT → EMP	0.463	11.935	0.000	0.290		Supported

Note: β = standardized path coefficient; t = bootstrap t-statistic; *p* = significance level based on 5000 bootstrap resamples; f^2^ = effect size; R^2^ = coefficient of determination for endogenous constructs. “Supported” indicates that the proposed hypothesis was statistically supported at the 95% confidence level.

**Table 6:** Indirect effects via narrative transportation.

Indirect Path	β	t	*p*-Value	Result
PIN → NT → EMP	0.139	5.576	0.000	Significant
PNA → NT → EMP	0.176	5.689	0.000	Significant
PIG → NT → EMP	0.050	2.116	0.034	Significant

Note: β = standardized indirect effect; t = bootstrap t-statistic; *p*-value = significance level based on 5000 bootstrap resamples. “Significant” indicates that the indirect effect through narrative transportation is statistically supported.

**Table 7:** Calibration anchors for the fsQCA analysis of high overall empathy.

Variable	Full Nonmembership (5%)	Crossover Point (50%)	Full Membership (95%)
PIN	−1.718	0.023	1.628
PNA	−1.585	0.028	1.640
PIG	−1.706	−0.052	1.832
NT	−1.766	0.062	1.713
EMP	−1.568	−0.031	1.634

**Table 8:** Analysis of necessary conditions for overall empathy.

Variable	EMP		~EMP	
	Consistency	Coverage	Consistency	Coverage
PIN (~PIN)	0.805 (0.528)	0.794 (0.544)	0.550 (0.788)	0.534 (0.800)
PNA (~PNA)	0.814 (0.507)	0.819 (0.512)	0.509 (0.817)	0.504 (0.812)
PIG (~PIG)	0.810 (0.523)	0.807 (0.533)	0.535 (0.803)	0.525 (0.806)
NT (~NT)	0.829 (0.519)	0.840 (0.520)	0.513 (0.839)	0.512 (0.828)

Note: The tilde sign “~” indicates the negation of the condition.

**Table 9:** Results of Configuration Analysis for high overall empathy.

Configuration	1	2	3
PIN	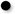	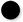	
PNA	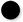		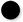
PIG		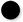	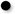
NT	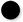	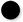	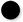
Consistency	0.915	0.916	0.921
Raw coverage	0.670	0.646	0.657
Unique coverage	0.055	0.031	0.043
Overall solution coverage	0.744		
Overall solution consistency	0.900		

Note: Large circles indicate core conditions and small circles peripheral conditions. Black circles (“●”) indicate the “presence” of a condition, and blank spaces in the solutions indicate “don’t care”. Core and peripheral conditions are distinguished based on the comparison between parsimonious and intermediate solutions.

**Table 10:** Calibration anchors for the fsQCA analysis of high empathy subdimensions.

Variable	Full Nonmembership (5%)	Crossover Point (50%)	Full Membership (95%)
PIN	−1.718	0.023	1.628
PNA	−1.586	0.030	1.640
PIG	−1.705	−0.052	1.833
NT	−1.762	0.062	1.713
AFE	−1.637	0.102	1.605
CE	−1.584	−0.009	1.565
ASE	−1.613	−0.062	1.693

**Table 11:** Results of Configuration Analysis for high empathy subdimensions.

	AFE	CE		ASE	
Configuration	1	1	2	1	2
PIN	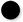	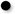		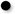	
PNA	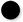	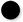	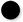	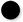	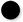
PIG	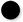		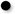		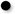
NT	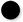	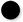	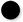	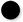	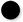
Consistency	0.878	0.903	0.907	0.907	0.917
Raw coverage	0.608	0.655	0.641	0.655	0.646
Unique coverage	0.608	0.058	0.044	0.051	0.041
Overall solution coverage	0.608	0.699		0.696	
Overall solution consistency	0.878	0.899		0.900	

Note: Large circles indicate core conditions and small circles peripheral conditions. Black circles (“●”) indicate the “presence” of a condition, and blank spaces in the solutions indicate “don’t care”. Core and peripheral conditions are distinguished based on the comparison between parsimonious and intermediate solutions.

## Data Availability

The data presented in this study are available upon request from the corresponding author.

## Abbreviations

The following abbreviations are used in this manuscript:

OMMG	Online Murder Mystery Game
PIN	Perceived interactivity
PNA	Perceived narrativity
PIG	Perceived integrity
NT	Narrative transportation
AFE	Affective empathy
CE	Cognitive empathy
ASE	Associative empathy

## Appendix A

### Appendix A.1

The complete questionnaire used in this study is presented in both English and Chinese in Table A1. The measurement items were adapted from previously validated scales, and the corresponding source references are provided for each construct.

**Table A1:** Measures.

Construct	Item	English and Chinese	Source
Perceived interactivity	PIN1	I have a good interactive experience with other online players.	(Gu et al., 2022)
		我与其他在线玩家之间有良好的互动游戏体验。	
	PIN2	During the online murder mystery game experience, I am able to effectively access narrative content.	
		在线剧本杀的体验过程中，我能够有效获取剧情内容。	
	PIN3	During the online murder mystery game experience, I can easily access information about the character I am playing.	
		在线剧本杀的体验过程中，我能轻松查看我所扮演角色的相关信息。	
Perceived narrativity	PNA1	The narrative plot of the online murder mystery game effectively conveys its underlying narrative motivations.	
		在线剧本杀的叙事情节，能够有效呈现其内在的叙事动机。	
	PNA2	The narrative plot of the online murder mystery game evokes emotional resonance and reflection in me.	
		在线剧本杀的叙事情节引发了我的情感共鸣与反思。	
	PNA3	The narrative plot of the online murder mystery game has a clear beginning, development, and ending.	
		在线剧本杀的叙事情节，具有清晰的开端、发展与结局。	
	PNA4	The narrative plot of the online murder mystery game tells a specific and unique story.	
		在线剧本杀的叙事情节，讲述了一个具体且独特的故事。	
Perceived integrity	PIG1	The online murder mystery game presents a complete storyline to me.	
		在线剧本杀向我呈现了一个完整的故事情节。	
	PIG2	The online murder mystery game presents a comprehensive storyline to me.	
		在线剧本杀向我呈现了全面详尽的故事情节。	
	PIG3	The online murder mystery game presents rich and diverse storylines to me.	
		在线剧本杀向我讲述了丰富的故事脉络。	
	PIG4	I can understand all the storylines in the online murder mystery game.	
		我能够理解在线剧本杀中的所有故事线索。	
Narrative transportation	NT1	I could easily imagine myself in the scene of the storylines unfolding in the online murder mystery game.	(Sherrick, 2021)
		我能够轻松地想象自己身处在线剧本杀的故事情节场景之中。	
	NT2	I have a vivid and concrete impression of the character I portray in the online murder mystery game.	
		我对自己所扮演的在线剧本杀角色有生动而具体的印象。	
	NT3	I am eager to know the outcome of the story in the online murder mystery game.	
		我渴望知道在线剧本杀中故事的结局。	
	NT4	I can approach the storylines in the online murder mystery game from different perspectives.	
		我能从不同视角代入在线剧本杀的故事情节。	
Affective empathy	AFE1	I believe that the emotions of the characters in the online murder mystery game are authentic.	(Augello, 2022)
		我相信在线剧本杀中各角色的情感是真实的。	
	AFE2	I experience the same emotions as the character I portray.	
		我体验到了与我所扮演角色相同的情感。	
	AFE3	My emotional state is similar to that of the character I portray.	
		我的情绪状态与我所扮演角色相似。	
	AFE4	I am able to perceive the emotions of other characters in the online murder mystery game.	
		我能够感知在线剧本杀中其他角色的情感。	
Cognitive empathy	CE1	During the online murder mystery game experience, I am able to think from the character’s perspective.	
		体验在线剧本杀的过程中，我能够以角色视角进行思考。	
	CE2	During the online murder mystery game experience, I can understand the situations of the characters.	
		体验在线剧本杀的过程中，我能够理解角色处境。	
	CE3	During the online murder mystery game experience, I can understand what the characters go through.	
		体验在线剧本杀的过程中，我能够理解角色经历。	
	CE4	During the online murder mystery game experience, I can understand the characters’ reactions and the choices they make.	
		体验在线剧本杀的过程中，我能够理解角色的反应以及其所做出的选择。	
Associative empathy	ASE1	During the online murder mystery game experience, I am immersed in the story of the character I portray.	
		体验在线剧本杀时，我沉浸在自己所扮演角色的故事之中。	
	ASE2	During the online murder mystery game experience, I empathize with what the character I portray experiences.	
		体验在线剧本杀时，我对我所扮演角色的经历产生了共鸣。	
	ASE3	I can identify with the storylines in the online murder mystery game.	
		我能对在线剧本杀中的故事情节产生认同。	
	ASE4	During the online murder mystery game experience, I can relate to the characters in the script.	
		体验在线剧本杀时，我对剧本里的角色感同身受。	

### Appendix A.2

To examine the robustness of the fsQCA results, additional analyses were conducted using alternative calibration anchors (10th, 50th, and 90th percentiles) instead of the original calibration thresholds (5th, 50th, and 95th percentiles). The following tables present the recalibrated calibration anchors, necessary condition analyses, and configurational solutions for both the overall empathy model and the three empathy subdimensions.

**Table A2:** Alternative calibration anchors for the overall empathy model.

Variable	Full Nonmembership (10%)	Crossover Point (50%)	Full Membership (90%)
PIN	−1.425	0.023	1.334
PNA	−1.342	0.028	1.372
PIG	−1.237	−0.052	1.371
NT	−1.287	0.062	1.413
EMP	−1.202	−0.031	1.330

**Table A3:** Robustness analysis of necessary conditions for overall empathy.

Variable	EMP		~EMP	
	Consistency	Coverage	Consistency	Coverage
PIN (~PIN)	0.793 (0.474)	0.769 (0.487)	0.503 (0.763)	0.490 (0.787)
PNA (~PNA)	0.805 (0.454)	0.796 (0.457)	0.463 (0.795)	0.459 (0.804)
PIG (~PIG)	0.793 (0.457)	0.783 (0.462)	0.469 (0.781)	0.464 (0.792)
NT (~NT)	0.806 (0.464)	0.822 (0.454)	0.443 (0.826)	0.454 (0.811)

Note: The tilde sign “~” indicates the negation of the condition.

**Table A4:** Robustness analysis of configuration results for high overall empathy.

Configuration	1	2	3
PIN			
PNA			
PIG			
NT			
Consistency	0.900	0.905	0.909
Raw coverage	0.643	0.611	0.627
Unique coverage	0.062	0.029	0.045
Overall solution coverage	0.718		
Overall solution consistency	0.887		

Note: Large circles indicate core conditions and small circles peripheral conditions. Black circles (“●”) indicate the “presence” of a condition, and blank spaces in the solutions indicate “don’t care”. Core and peripheral conditions are distinguished based on the comparison between parsimonious and intermediate solutions.

**Table A5:** Alternative calibration anchors for the empathy subdimensions.

Variable	Full Nonmembership (5%)	Crossover Point (50%)	Full Membership (95%)
PIN	−1.424	0.023	1.334
PNA	−1.342	0.030	1.371
PIG	−1.236	−0.052	1.369
NT	−1.287	0.062	1.414
AFE	−1.149	0.102	1.337
CE	−1.312	−0.009	1.311
ASE	−1.232	−0.062	1.426

**Table A6:** Robustness analysis of necessary conditions for affective empathy.

Variable	AFE		~AFE	
	Consistency	Coverage	Consistency	Coverage
PIN (~PIN)	0.782 (0.486)	0.713 (0.469)	0.514 (0.722)	0.531 (0.789)
PNA (~PNA)	0.792 (0.468)	0.738 (0.443)	0.479 (0.751)	0.505 (0.804)
PIG (~PIG)	0.791 (0.471)	0.734 (0.447)	0.485 (0.747)	0.509 (0.801)
NT (~NT)	0.793 (0.478)	0.760 (0.439)	0.460 (0.779)	0.499 (0.810)

Note: The tilde sign “~” indicates the negation of the condition.

**Table A7:** Robustness analysis of necessary conditions for cognitive empathy.

Variable	CE		~CE	
	Consistency	Coverage	Consistency	Coverage
PIN (~PIN)	0.780 (0.486)	0.769 (0.507)	0.514 (0.759)	0.493 (0.770)
PNA (~PNA)	0.788 (0.475)	0.793 (0.486)	0.482 (0.789)	0.472 (0.783)
PIG (~PIG)	0.766 (0.479)	0.769 (0.491)	0.490 (0.763)	0.478 (0.761)
NT (~NT)	0.803 (0.481)	0.832 (0.477)	0.458 (0.833)	0.462 (0.804)

Note: The tilde sign “~” indicates the negation of the condition.

**Table A8:** Robustness analysis of necessary conditions for associative empathy.

Variable	ASE		~ASE	
	Consistency	Coverage	Consistency	Coverage
PIN (~PIN)	0.775 (0.486)	0.760 (0.504)	0.514 (0.751)	0.496 (0.766)
PNA (~PNA)	0.793 (0.466)	0.793 (0.473)	0.473 (0.790)	0.466 (0.789)
PIG (~PIG)	0.789 (0.468)	0.786 (0.477)	0.479 (0.782)	0.470 (0.785)
NT (~NT)	0.780 (0.487)	0.804 (0.481)	0.465 (0.806)	0.472 (0.783)

Note: The tilde sign “~” indicates the negation of the condition.

**Table A9:** Robustness analysis of configuration results for high empathy subdimensions.

	AFE	CE		ASE
Configuration	1	1	2	1
PIN				
PNA				
PIG				
NT				
Consistency	0.852	0.892	0.897	0.905
Raw coverage	0.575	0.627	0.609	0.617
Unique coverage	0.575	0.064	0.046	0.617
Overall solution coverage	0.575	0.673		0.617
Overall solution consistency	0.852	0.890		0.905

Note: Large circles indicate core conditions and small circles peripheral conditions. Black circles (“●”) indicate the “presence” of a condition, and blank spaces in the solutions indicate “don’t care”. Core and peripheral conditions are distinguished based on the comparison between parsimonious and intermediate solutions.

## References

[B1-behavsci-16-01520] Adornetti I., Deriu V., Chiera A., Altavilla D., Luciani F., Garello S., Ferretti F. (2026). The story effect: Identification as a key mediator of persuasion in climate change narratives. Frontiers in Communication.

[B2-behavsci-16-01520] Augello A. (2022). Unveiling the reasoning processes of robots through introspective dialogues in a storytelling system: A study on the elicited empathy. Cognitive Systems Research.

[B3-behavsci-16-01520] Chen Q., Huang X., Li S. (2026). Digital storytelling in VR museums: How interaction with museum treasures affects tourists’ responses?. Tourism Management.

[B4-behavsci-16-01520] Cheng K.-H. (2023). An epistemic curiosity-evoking model for immersive virtual reality narrative reading: User experience and the interaction among epistemic curiosity, transportation, and attitudinal learning. Computers & Education.

[B5-behavsci-16-01520] Cuadra A., Wang M., Stein L. A., Jung M. F., Dell N., Estrin D., Landay J. A. (2024). The illusion of empathy? Notes on displays of emotion in human-computer interaction. 2024 CHI Conference on Human Factors in Computing Systems.

[B6-behavsci-16-01520] Daneels R., Vandebosch H., Walrave M. (2023). “Deeper gaming”: A literature review and research agenda on eudaimonia in digital games research. Technology, Mind, and Behavior.

[B7-behavsci-16-01520] de Waal F., Preston S. D. (2017). Mammalian empathy: Behavioural manifestations and neural basis. Nature Reviews Neuroscience.

[B8-behavsci-16-01520] Eekhof L. S., van Krieken K., Sanders J., Willems R. M. (2023). Engagement with narrative characters: The role of social-cognitive abilities and linguistic viewpoint. Discourse Processes.

[B9-behavsci-16-01520] Felnhofer A., Wittmann L., Reichmann A., König-Teshnizi D., Kothgassner O. D. (2023). Character identification is predicted by narrative transportation, immersive tendencies, and interactivity. Current Psychology.

[B10-behavsci-16-01520] Golab D., Barbot B. (2024). Building narratives through empathy: The role of empathy mechanisms and associative thinking in creative writing. The Journal of Creative Behavior.

[B11-behavsci-16-01520] Green M. C., Frank L. B., Falzone P. (2021). Transportation into narrative worlds. Entertainment-education behind the scenes: Case studies for theory and practice.

[B12-behavsci-16-01520] Green M. C., Appel M., Gawronski B. (2024). Chapter one—Narrative transportation: How stories shape how we see ourselves and the world. Advances in experimental social psychology.

[B13-behavsci-16-01520] Green M. C., Brock T. C. (2000). The role of transportation in the persuasiveness of public narratives. Journal of Personality and Social Psychology.

[B14-behavsci-16-01520] Green M. C., Brock T. C., Kaufman G. F. (2004). Understanding media enjoyment: The role of transportation into narrative worlds. Communication Theory.

[B15-behavsci-16-01520] Green M. C., Jenkins K. M. (2014). Interactive narratives: Processes and outcomes in user-directed stories. Journal of Communication.

[B16-behavsci-16-01520] Gu C., Chen J., Yang C., Wei W., Jiang Q., Jiang L., Wu Q., Lin S.-Y., Yang Y. (2022). Effects of AR picture books on German teaching in universities. Journal of Intelligence.

[B17-behavsci-16-01520] Hadjipanayi C., Christofi M., Banakou D., Michael-Grigoriou D. (2024). Cultivating empathy through narratives in virtual reality: A review. Personal and Ubiquitous Computing.

[B18-behavsci-16-01520] Hair J. F., Risher J. J., Sarstedt M., Ringle C. M. (2019). When to use and how to report the results of PLS-SEM. European Business Review.

[B19-behavsci-16-01520] Hamby A., Kim H., Spezzano F. (2024). Sensational stories: The role of narrative characteristics in distinguishing real and fake news and predicting their spread. Journal of Business Research.

[B20-behavsci-16-01520] Hao X., Valayakkad Manikandan S., Demir E., Eyers D. (2025). Visual narratives and audience engagement: Edutainment interactive strategies with computer vision and natural language processing. Journal of Research in Interactive Marketing.

[B21-behavsci-16-01520] Henseler J., Dijkstra T. K., Sarstedt M., Ringle C. M., Diamantopoulos A., Straub D. W., Ketchen D. J., Hair J. F., Hult G. T. M., Calantone R. J. (2014). Common beliefs and reality about PLS: Comments on Rönkkö and Evermann (2013). Organizational Research Methods.

[B22-behavsci-16-01520] Henseler J., Ringle C. M., Sarstedt M. (2015). A new criterion for assessing discriminant validity in variance-based structural equation modeling. Journal of the Academy of Marketing Science.

[B23-behavsci-16-01520] Heravi B. (2025). Exploratory and explanatory features in data storytelling: Untangling the interplay and associations with linearity, interactivity, and structure. Journal of Computational Social Science.

[B24-behavsci-16-01520] Ho J. C. F., Wong A. L. L. (2026). Effects of witnessing accidents in virtual reality on reception of safety training: The role of empathy toward virtual victims. Safety Science.

[B25-behavsci-16-01520] Huajing Industry Research Institute (2025). Market scale, competitive pattern and development trend of China’s online script murder industry in 2025.

[B26-behavsci-16-01520] Huang C., Wu Z., Sha S., Liu C., Yang L., Jiang P., Zhang H., Yang C. (2025). The dark side of empathy: The role of excessive affective empathy in mental health disorders. Biological Psychiatry.

[B27-behavsci-16-01520] Jami P. Y., Walker D. I., Mansouri B. (2024). Interaction of empathy and culture: A review. Current Psychology.

[B28-behavsci-16-01520] Kang W., Shao B., Zhang Y. (2024). How does interactivity shape users’ continuance intention of intelligent voice assistants? Evidence from SEM and fsQCA. Psychology Research and Behavior Management.

[B29-behavsci-16-01520] Kock N. (2015). Common method bias in PLS-SEM: A full collinearity assessment approach. International Journal of e-Collaboration (IJeC).

[B30-behavsci-16-01520] Koenitz H. (2023). Understanding interactive digital narrative: Immersive expressions for a complex time.

[B31-behavsci-16-01520] Li Y., Sun M. (2025). The impact of perceived interactivity on purchase intention in interactive video: A CAB-based chain mediation model of empathy, immersion, and arousal. Frontiers in Communication.

[B32-behavsci-16-01520] Liang S., Chen M., Toups Dugas P. O., Smith G., Bohrer R. (2025). The collaborative sensemaking play of jubensha games: A deconstruction, taxonomy, and analysis. ACM Games: Research and Practice.

[B33-behavsci-16-01520] Liu J.-X. (2023). The influence of narrative transportation on university students’ environmental intentions: A serial mediation of empathy with nature and environmental Attitudes. Journal of Cleaner Production.

[B34-behavsci-16-01520] Liu T., Giorgi S., Aich A., Lahnala A., Curtis B., Ungar L., Sedoc J. (2025). The illusion of empathy: How AI chatbots shape conversation perception. Proceedings of the AAAI Conference on Artificial Intelligence.

[B35-behavsci-16-01520] Liu Y. (2023). Experiencing China’s intangible cultural heritage in role-playing games: Comparative studies between MMORPGs and larps. International Journal of Role-Playing.

[B36-behavsci-16-01520] Lu A. S., Moller A. C. (2026). Elaborating the role of narrative and self-determination theory in video game design research. Interacting with Computers.

[B37-behavsci-16-01520] Meakin E. (2024). Video game structural layers for narrative design and articulation. Digital Creativity.

[B38-behavsci-16-01520] Mehrab Ashrafi D., Mone F. H., Zabeen M., Sarker M. A. R., Shahid T. (2025). What drives users to recommend mobile fitness apps? A three-stage analysis using PLS-SEM, machine learning, and fsQCA. International Journal of Human–Computer Interaction.

[B39-behavsci-16-01520] Nell V., Nell V. (1988). Lost in a book: The psychology of reading for pleasure.

[B40-behavsci-16-01520] Ng P. (2025). Sino-virtuality: Transcoding Chinese aesthetic principles to Jubensha’s immersive economies. Cogent Arts & Humanities.

[B41-behavsci-16-01520] Nonomura R., Mori H. (2025). Who speaks next? Multi-party AI discussion leveraging the systematics of turn-taking in Murder Mystery games. Frontiers in Artificial Intelligence.

[B42-behavsci-16-01520] Pappas I. O., Woodside A. G. (2021). Fuzzy-set qualitative comparative analysis (fsQCA): Guidelines for research practice in information systems and marketing. International Journal of Information Management.

[B43-behavsci-16-01520] Podsakoff P. M., MacKenzie S. B., Lee J.-Y., Podsakoff N. P. (2003). Common method biases in behavioral research: A critical review of the literature and recommended remedies. Journal of Applied Psychology.

[B44-behavsci-16-01520] Polydorou D. (2024). Immersive storytelling experiences: A design methodology. Digital Creativity.

[B45-behavsci-16-01520] Preston S. D., Ermler M., Lei Y., Bickel L. (2020). Understanding empathy and its disorders through a focus on the neural mechanism. Cortex.

[B46-behavsci-16-01520] Ragin C. C. (2009). Redesigning social inquiry: Fuzzy sets and beyond.

[B47-behavsci-16-01520] Roth C. (2019). The ‘Angstfabriek’ experience: Factoring fear into transformative interactive narrative design. International Conference on Interactive Digital Storytelling.

[B48-behavsci-16-01520] Ryan M.-L. (2006). Avatars of story.

[B49-behavsci-16-01520] Ryan M.-L. (2015). Narrative as virtual reality 2: Revisiting immersion and interactivity in literature and electronic media.

[B50-behavsci-16-01520] Sarstedt M., Hair J. F., Cheah J.-H., Becker J.-M., Ringle C. M. (2019). How to specify, estimate, and validate higher-order constructs in PLS-SEM. Australasian Marketing Journal.

[B51-behavsci-16-01520] Shen L. (2010). On a scale of state empathy during message processing. Western Journal of Communication.

[B52-behavsci-16-01520] Sherrick B. (2021). Empirically comparing flow, narrative engagement, and enjoyment as responses to a computer game. Atlantic Journal of Communication.

[B53-behavsci-16-01520] Shmueli G., Sarstedt M., Hair J. F., Cheah J.-H., Ting H., Vaithilingam S., Ringle C. M. (2019). Predictive model assessment in PLS-SEM: Guidelines for using PLSpredict. European Journal of Marketing.

[B54-behavsci-16-01520] Sukhov A., Friman M., Olsson L. E. (2023). Unlocking potential: An integrated approach using PLS-SEM, NCA, and fsQCA for informed decision making. Journal of Retailing and Consumer Services.

[B55-behavsci-16-01520] Szanto T., Krueger J. (2019). Introduction: Empathy, shared emotions, and social identity. Topoi.

[B56-behavsci-16-01520] Thomas V. L., Grigsby J. L. (2024). Narrative transportation: A systematic literature review and future research agenda. Psychology & Marketing.

[B57-behavsci-16-01520] Toh W. (2023). The player experience and design implications of narrative games. International Journal of Human–Computer Interaction.

[B58-behavsci-16-01520] Tseng C.-I., Thiele L. (2024). Actions and digital empathy in the interactive storytelling of serious games: A multimodal discourse approach. Social Semiotics.

[B59-behavsci-16-01520] Turner B. R. (2025). Narrative affordances: What stories can and cannot do. Academy of Management Annals.

[B60-behavsci-16-01520] Tzeng S.-Y., Huang K. J., Wong W. M. (2025). From screen to plate: The role of narrative transportation in shaping destination food perceptions. Asia Pacific Journal of Marketing and Logistics.

[B61-behavsci-16-01520] Wang F., Cheung A. C. K., Chai C. S., Liu J. (2025). Development and validation of the perceived interactivity of learner-AI interaction scale. Education and Information Technologies.

[B62-behavsci-16-01520] Wang J. (2025). Integrating virtual and augmented reality in storytelling: Exploring the evolution of narratives, audience engagement, and technological accessibility. International Journal of Inclusive Education.

[B63-behavsci-16-01520] Wu D., Shi H., Sun Z., Liu B. (2024). Deciphering digital detectives: Understanding LLM Behaviors and capabilities in multi-agent mystery games.

[B64-behavsci-16-01520] Wu X., Zhang M., Shi S. (2024). Understanding customers’ interactive experience in immersive performing art: A narrative transportation perspective. Nankai Business Review International.

[B65-behavsci-16-01520] Xiong S., Wen R., Zheng H. (2023). Player category research on murder mystery games. International Journal of Role-Playing.

[B66-behavsci-16-01520] Yu Y., Zhang H. (2023). The impact of text narrative elements in mobile murder mystery games on immersive experience. 2023 International Conference on Culture-Oriented Science and Technology (CoST).

[B67-behavsci-16-01520] Yuan J., Shah S. K., Li Z. (2024). Consumers’ adoption intention to Metaverse applications: An exploration through fsQCA approach. Journal of Consumer Behaviour.

[B68-behavsci-16-01520] Zhang J., Quoquab F., Mohammad J. (2025). “Do video game players dream of metaverse traveling?” The role of gamification technology and game immersion experience. Tourism Review.

[B69-behavsci-16-01520] Zhou C., Kim S. (2023). Testing the effect of an interactive narrative: The mediating role of transportation and the moderating roles of narrative ending and issue involvement. Health Communication.

